# Designing universal primers for the isolation of DNA sequences encoding Proanthocyanidins biosynthetic enzymes in *Crataegus aronia*

**DOI:** 10.1186/1756-0500-5-427

**Published:** 2012-08-10

**Authors:** Afnan Saeid Zuiter, Jammal Sawwan, Ayed Al Abdallat

**Affiliations:** 1 Department of Horticulture and Crop Science, Faculty of Agriculture, University of Jordan, Amman 11942, Jordan; 2 Hamdi Mango Center for Scientific Research, University of Jordan, Amman 11942, Jordan

**Keywords:** Alignment PCR Analysis, BLAST, Multiple sequence alignment, Proanthocyanidins, Rosaceae

## Abstract

**Background:**

Hawthorn is the common name of all plant species in the genus *Crataegus*, which belongs to the *Rosaceae* family. *Crataegus* are considered useful medicinal plants because of their high content of proanthocyanidins (PAs) and other related compounds. To improve PAs production in *Crataegus* tissues, the sequences of genes encoding PAs biosynthetic enzymes are required.

**Findings:**

Different bioinformatics tools, including BLAST, multiple sequence alignment and alignment PCR analysis were used to design primers suitable for the amplification of DNA fragments from 10 candidate genes encoding enzymes involved in PAs biosynthesis in *C. aronia*. DNA sequencing results proved the utility of the designed primers. The primers were used successfully to amplify DNA fragments of different PAs biosynthesis genes in different *Rosaceae* plants*.*

**Conclusion:**

To the best of our knowledge, this is the first use of the alignment PCR approach to isolate DNA sequences encoding PAs biosynthetic enzymes in *Rosaceae* plants.

## Background

Hawthorn (*Crataegus spp*.) is a member of the *Rosaceae* family, belonging to the *Spiraeoideae* subfamily in the *Pyroidae* supertribe in the *Pyreae* subtribe [1]. There are approximately 280 species in the genus *Crataegus*, which are widely distributed across the Mediterranean, North Africa, Europe, Central and Eastern Asia and North America [2]. In the eastern Mediterranean region, the predominant *Crataegus* species is *C. aronia* L., which is found on dry hillsides, mountains and in areas receiving more than 300 mm annual rainfall [3].

In folk medicine, a decoction of *Crataegus* leaves and unripe fruits has been used to treat disorders such as cardiovascular diseases, cancer, diabetes, and impotence [2,4]. The medicinal properties of *Crataegus* are primarily related to its antioxidant activities and the reduction of free radical-induced oxidative stress [5,6], mediated by secondary metabolites, such as flavonoids, oligomeric Proanthocyanidins (PAs), ethanobotanical and ethanopharmacological compounds [7].

PAs, also known as condensed tannins, are polyphenolic compounds formed as a branch of the flavonoid biosynthetic pathway [8]. PAs are found in the fruits, bark, leaves and seeds of many plants including *Crataegus*[2,9] and their function include defense against herbivores and resistance to abiotic stresses [9]. They are also important quality components of many fruits, providing the flavor and color to beverages [10]. The PAs biosynthesis pathway and its regulatory genes have been dissected genetically and biochemically in a number of plants [11] including *Arabidopsis*[12,13]*Vitis vinifera*[10,14,15], bilberry (*Vaccinium myrtillus*) [16], soybean (*Glycine max*) [17] and persimmon (*Diospyros kaki*) [18], but not in *Crataegus*. Therefore, there is a need to identify DNA sequences encoding phenylpropanoid biosynthesis enzymes in *Crataegus*. In this study, bioinformatics tools were used to design specific primers to amplify DNA sequences of key genes encoding enzymes involved in the PAs biosynthetic pathway in *C. aronia*.

## Findings

### Designing of Primers for the Isolation of PAs Biosynthetic genes

First, DNA sequences encoding PAs biosynthetic enzymes from four different *Pyreae* subtribe genera were retrieved from the NCBI databases, which resulted in several DNA sequences representing ten selected PAs biosynthesis enzymes (Additional file 1: Table S1). All the DNA sequences were complete coding regions, except for the *4-Cl* gene. BLAST searching with the selected DNA sequences retrieved related DNA sequences from different plant, including DNA sequences encoding PAs biosynthetic enzymes from the *Spiraeoideae*, *Rosaceae*, *Rosales* and *Fabids* taxa (data not shown). All retrieved sequences were analyzed for their nucleic acid type (genomic DNA or mRNA or EST), the presence or absence of introns, their length (partial or full length) and for their redundancy in different nucleotide databases.

Overlapping partial DNA sequences were gathered and assembled into contigs using the VectorNTI software. Finally, the DNA sequences of each PAs biosynthetic gene were grouped according to their taxon and then used to design universal primers using alignment PCR analysis in the VectorNTI software.

To design of PAs biosynthesis genes specific primers, multiple sequence alignment (MSA) analysis was performed initially among all retrieved DNA sequences from selected taxa using ClustalW in the Vector NTI suite. The MSA results were inspected and filtered to exclude DNA sequences with low levels of similarity and wide gaps. Secondary and tertiary MSAs were performed on the most similar DNA sequences until the aligned sequences showed large areas of sequence similarity. Additional file 2 shows the *ANS* genes’ alignment as an example. In general, conserved regions in the DNA sequences of PAs biosynthetic enzymes were identified in groups of closely related genera in the *Rosaceae* family, with some exceptions (data not shown). Alignment PCR analysis was then performed and the most suitable primers for PCR amplification of targeted genes in *Rosaceae* plants were selected and subjected to further analysis (Table 1).

**Table 1:** **Characteristics of the designed primers specific for *Rosaceae *PAs biosynthesis genes used in this study**

**Gene name**	**Primer**	**Primer sequence (5' → 3')**	**T**_**m**_**°C**^**a**^
*ANR*	ANRFwd1	CCCACCTMACAGCACTACAA ^b^	53.8
	ANRRev1	TACCGACCAGAAGCAGATTC	51.8
	ANRFwd2	GGAGTGATTTGGAGTTCTTG	49.7
	ANRRev2	GGAAAATCTCCAAACTCAGT	47.7
*ANS*	ANSFwd1	TTTGAYCTTCCCATTGAGCA	49.7
	ANSRev1	GCATTTTGGGTAGTAGTTGA	47.7
	ANSFwd2	CAGCTTGAGTGGGAGGAYTA	53.8
	ANSRev2	GCAGGCCRGGAACCATGTTG	57.9
*4-CL*	4-CLFwd1	CCTGGAGAGATTTGCATCAG	51.8
	4-CLRev1	AATACTCGATTTATTCTTTTATA	42.8
	4-CLFwd2	ATCATGAAAGGTTAYCTTAATG	45.5
	4-CLRev2	GGAATGGCTTCGATGAAAAA	47.7
*CHI*	CHIFwd1	AGGGGGTYGGAGATTCAGGG	55.9
	CHIRev1	GGGAAGKTTTGATCYTTGAA	45.6
	CHIFwd2	TTCGTGAAGTTCACGGCGAT	51.8
	CHIRev2	TGGGAAGGTCTGATCTTTGA	49.7
*C4H*	C4HFwd1	CTCCGYATGGGRCAGCGCAA	60
	C4HRev1	TTGTTGTACATCATMAGCTG	47.7
	C4HFwd2	CAGCTBATGATGTACAACAA	47.7
	C4HRev2	TGGATTTCRGGGTGGTTCAC	53.8
*CHS*	CHSFwd1	AAGGCCATYAAGGAATGGGG	53.8
	CHSRev1	AATGTRAGCCCDACTTCACG	53.8
	CHSFwd2	AAGGAATGGGGHCAGCCCAA	55.9
	CHSRev2	CTCACYTCRTCCAAAATAAA	47.7
*DFR*	DFRFwd1	CCTGACGCTGTGGAAGGCGG	60
	DFRRev1	TAATGGTRATGAAATCAATG	43.6
	DFRFwd2	TGCASCGGAGTGTTYCATGT	53.8
	DFRRev2	TGAGGCTTGGTGGCATRGATGG	58.6
*LAR*	LARFwd1	GTTCGTMGCYGAAGCCAGCC	60
	LARRev1	TGGAACYGATCCAACGGTGG	55.9
	LARFwd2	AGGGCYRATCCGGTKGAACC	60
	LARRev2	AGGGTSCGRCCAATTTTCTT	51.8
*F3H*	F3HFwd1	GACATGTCCGGYGGCAAAAAGGG	60.6
	F3HRev1	CTCATCTTCTTCTTGTACAT	45.6
	F3HFwd2	ACGTGGATCACCGTTCAACC	53.8
	F3HRev2	GGTTGAACGGTGATCCACGT	53.8
*PAL*	PALFwd1	ATCGATGTTTCRAGGAACAA	47.7
	PALRev1	AAAGAGTTAACATCTTGGTT	43.6
	PALRev2	AAAAATGTRGAAGACATGAG	45.6

^a^ Melting temperature.

^b^ Degenerate nucleotides codes where B (C/G/T), D (A/G/T), H (A/C/T), K (G/T), M (A/C), N (A/C/G/T), R (A/G), S (G/C), V (A/C/G), W (A/T), Y (C/T).

To validate the alignment PCR results, the primers were BLAST searched against the nucleotide databases of the Genome Database for *Rosaceae* ( http://www.rosaceae.org) and against the NCBI GenBank nucleotide databases of flowering plants. As expected, the percentage of similarity between the designed primers and the DNA sequences of the targeted PAs genes in the *Rosaceae* family was very high, with minor exceptions for *Malus*, *Prunus* and *Fragaria* spp. (see Additional file 3). In addition, many of the primers were conserved in different flowering plants, including grape, soybean, arabidopsis and poplar (data not shown).

However, some low similarity (one or two mismatches) between the designed primers and the DNA sequences of the targeted PAs genes in some *Rosaceae* family members were observed (data not shown). To resolve this, degenerate nucleotides were included in the primer sequences to ensure their functionality with different plants from the *Rosaceae* family (Table 1).

### Testing the designed primers using RT-PCR in different *Rosaceae* plants

**Figure 1:**
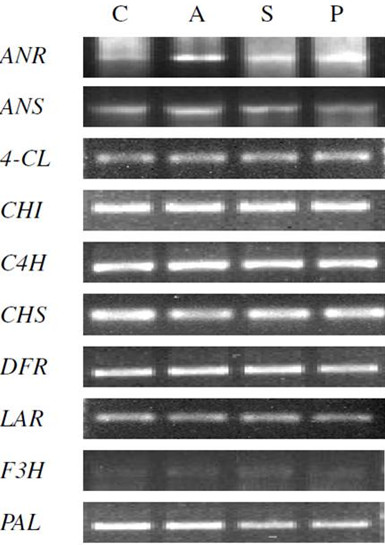
**Semi-quantitative RT-PCR expression patterns of the PAs biosynthesis genes: *ANR, ANS, 4-CL, CHI, CHS*, *DFR*, *LAR*, *F3H * and *PAL *in *Crataegus aronia *callus (C), apple (A), strawberry (S) and peach (P).** Amplification products were separated on a 1% agarose gel. The primers combinations for each gene are indicated in additional file 4.

The efficiency of the designed primers in amplifying DNA fragments of the corresponding PAs biosynthesis genes from *C. aronia* was tested using RT-PCR. In general, DNA fragments of *C. aronia* PAs biosynthesis genes were successfully amplified using cDNA prepared from *C. aronia* callus cells and different combinations of designed primers (Additional file 4; Figure 1). Successful primer combinations produced PCR amplicons from *C. aronia* cDNA of the expected size of the corresponding DNA fragments in the target gene.

DNA sequencing of the positive amplicons confirmed the results of the RT-PCR analysis (data not shown). The sequencing data were further analyzed using bioinformatics tools, such as BLAST searching against the Genome Database for *Rosaceae* ( http://www.rosaceae.org) and the NCBI GenBank nucleotide databases of flowering plants. DNA sequencing results and bioinformatics analysis of the amplicons confirmed that the PCR products were DNA fragments of PAs biosynthesis genes from *C. aronia*.

To test designed primers’ ability to amplify PCR products in different *Rosaceae* plants, the most successful primer combinations in *C. aronia* (Additional file 4) were tested using cDNA prepared from leaf samples from apple, strawberry and peach. The designed primers successfully amplified PCR products from cDNA prepared from *C. aronia* callus tissue, apple, strawberry and peach (Figure 1).

### Discussion

To gain better understanding of the PAs biosynthesis pathway and the mechanism governing their synthesis in plants, several genes encoding PAs biosynthetic enzymes have been isolated [10-19]. Metabolic and genetic engineering systems have used this genetic information to boost the production of PAs in different plants [20,21]. In the present study, alignment PCR was used to design functional primers for genes encoding PAs biosynthetic enzymes in *Rosaceae* plants. Partial DNA sequences were obtained for 10 targeted genes encoding PAs biosynthetic enzymes from *C. aronia* using the designed primers. Many of the designed primers are conserved across different plant species, indicating that they could be used to obtain genes encoding PAs biosynthetic enzymes in non-related organism. The conserved primers might be used in colinearity and comparative genomics studies between highly related species [22].

The alignment PCR approach easily, specifically and effectively produced DNA sequences from the targeted genes in different plant species. Degenerate primers, rapid amplification of cDNA ends (RACE) primer technology and PCR-based walking strategies were used to characterize PAs biosynthetic enzyme genes *ANR, ANS, F3H, DFR, LAR and FS* in strawberry [23]. In that study, the degenerate primers were designed using MSA based on protein sequences. The same strategy was used to amplify full-length coding sequences of the *ANR* and *LAR* genes from grape [10].

Compared with the alignment PCR analysis, the degenerate primers approach is based on extracting DNA sequences from conserved amino acids in homologous proteins [24]. The degenerate primers are designed to amplify related but not identical DNA sequences. Such an approach might result in lower specificity of the designed primers and increase probability of producing non-targeted amplicons [24,25].

Several public databases have been developed for designing universal primers for a particular gene across different taxa, e.g. UniPrime [26], and UniPrime2 [27]. The alignment PCR protocol described in this study adapts the same principles, parameters and approaches for universal primers design described in the public databases. However, such databases are not suited for partial CDS sequences that are difficult to handle in MSA analysis. In this study, several partial DNA sequences retrieved from EST databases were subjected to contig assembly to produce full-length DNA sequences that were easier to analyze using MSA. The contig assembly of partial CDS approach could be used to improve currently existing public universal primers databases.

### Conclusions

In this study, genetic information related to 10 different genes encoding PAs biosynthetic enzymes from *C*. *aronia* plant were obtained. Such information can be used to clone the full-length gene sequences from *C. aronia*. The information can also be used to improve PAs production in *C. aronia* using genetic engineering and tissue culture systems developed specifically for this plant species [28].

The designed primers showed high levels of sequence similarity with their corresponding genes in different *Rosaceae* plants; therefore, they could be used to isolate DNA fragments of PAs genes from different *Rosaceae* plants.

### Methods

#### Plant material

Callus cultures of *C. aronia* were established as described previously [28]. To test the functionality of the identify primers with other *Rosaceae* species, leaf samples from apple (*Malus domestica*), peach (*Prunus persica*) and strawberry (*Fragaria x ananassa*) were used.

#### Bioinformatics analysis

First, DNA or protein sequences of the targeted PAs biosynthetic enzymes in *Crataegus* and closely related plant species and genera were retrieved from NCBI GenBank databases (Additional file 1: Table S1). The sequences were subjected to different BLAST algorithms to gather DNA sequences of the corresponding PA biosynthesis from different plant taxa, especially the *Pyreae* subtribe, *Pyrodae* supertribe, *Spiraeoideae* subfamily, *Rosaceae* family, *Rosales* order and *Fabids*. The obtained BLAST results were filtered and the best hits (expectation value (ΔE) less than 1e-50) were chosen for alignment PCR analysis. The retrieved DNA sequences were subjected to MSA analysis using the ClustalW program in the Vector NTI™ Suite version 11.5 (Invitrogen, Carlsbad, CA, USA). The MSA results were used to design taxa specific primers based on conserved DNA sequences, using alignment PCR and ‘primer design’ tools, as described in the VectorNTI Suite version 11.5 manual. Finally, the best primers combinations were selected for the PCR analysis.

#### RNA extraction and reverse transcription PCR

Callus cells of *C*. *aronia* and leaf samples of different *Rosaceae* plants were used to extract total RNA using the EZ-10 Spin Column Total RNA Mini-Preps Super Kit (Biobasic Inc., Ontario, Canada), following the manufacturer’s instructions. For reverse transcription (RT) -PCR analysis, 1 μg of the isolated total RNA samples were converted into first-strand cDNA using the GoScript^TM^ reverse transcription kit (Promega, Madison, Wisconsin), following the manufacturer’s instructions. The synthesized cDNAs were used as PCR templates to amplify DNA fragments encoding PAs biosynthetic enzymes. The PCR was performed in an Applied Biosystems 9700 thermocycler (Applied Biosystems, Carlsbad, CA, USA) using the i-MAXII PCR Master Mix solution (iNtRON Biotechnology, Seoul, Korea) and different primer combinations (Table 1). The 25 μl reactions contained 2.5 μl of the synthesized cDNA, 0.5 μM of each primer and 12.5 μl of i-MAXII solution, following the manufacturer's instructions. The PCR conditions were 94°C for 5 min; 35 cycles of 94°C for 45 seconds, 50–60°C (depending on the primer combination) for 30 seconds, and 72°C for 1 minute; and a final extension at 72°C for 10 minutes. The PCR products were separated on 1.5% agarose gels and visualized using the Safe Red stain (iNtRON Biotechnology, Seoul, Korea) using Gel Doc™ XR + (BioRad, Hercules, CA). DNA fragments of the expected sizes were excised from the agarose gel and extracted using the Wizard® SV Gel and PCR Clean-up System (Promega, Madison, Wisconsin), following the manufacturer’s instructions. The DNA fragments were sequenced using an ABI 3730XL DNA sequencer by Macrogen Inc. (Seoul, Korea).

## Abbreviations

ANR: Anthocyanidin reductase;ANS: Anthocyanidin synthase;C4H: Cinnamate 4-hydroxylase;CHI: Chalcone isomerase;CHS: Chalcone synthase;4-CL: 4-Coumarate:coenzyme A ligase;DFR: Dihydroflavonol reductase;F3H: Flavonoid 3-hydroxylase;LAR: Leucoanthocyanidin reductase;MSA: Multiple sequence alignment;PAL: Phenylalanine ammonia lyase;PAs: Proanthocyanidins.

## Competing interests

The authors declare that they have no competing interests.

## Authors’ contributions

ASZ performed most of the experimental work and bioinformatics analysis; JSS developed the conceptual aspects of the work and edited the manuscript; AMA conceived the research, helped in the molecular and bioinformatics work and drafted the manuscript; all authors read and approved the final manuscript.

## Supplementary Material

Additional file 1: Table S1 GenBank accession numbers of reference genes encoding *Pyreae* PAs biosynthesis enzymes used in this study. Click here for file

Additional file 2 ** Multiple sequence alignment analysis of the *ANS *gene showing the position of the *ANS *designed primers (underlined) used in this study.** Click here for file

Additional file 3 ** Pairwise sequence alignment between the designed primers and the blast retrieved DNA sequences encoding PAs biosynthesis from *Malus***, ***Prunus *and *Fragaria *spp. using the NCBI GenBank nucleotide (nr/nt) and est databases.** Click here for file

Additional file 4 ** Estimated sizes of PCR amplicons of the PAs biosynthesis gene resulted from using different primers combinations and cDNA prepared from *Crataegus *callus.** Click here for file
