# Supplementary material for: Designing universal primers for the isolation of DNA sequences encoding Proanthocyanidins biosynthetic enzymes in Crataegus aronia
Source: BMC Res Notes. 2012 Aug 10;5:427. doi: 10.1186/1756-0500-5-427 (PMC3492024; doi:10.1186/1756-0500-5-427)
Supplement: Additional file 1: Table S1 — GenBank accession numbers of reference genes encoding Pyreae PAs biosynthesis enzymes used in this study. [file 1756-0500-5-427-S1.docx]

Table 1. GenBank accession numbers of reference genes encoding *Pyreae* PAs biosynthesis enzymes used in this study.

| Gene name | Organism | Accession number | CDS nature |
| --- | --- | --- | --- |
| *ANR* | *Malus x domestica* | AY830130 | Complete |
|  |  |  |  |
| *ANS* | *Pyrus communis* | [DQ230994](http://www.ncbi.nlm.nih.gov/entrez/query.fcgi?cmd=Retrieve&db=Nucleotide&list_uids=81295651&dopt=GenBank&RID=D4TR97F3016&log$=nucltop&blast_rank=1) | Complete |
|  |  |  |  |
| *4-Cl* | *Eriobotrya japonica* | EF685345 | Partial |
|  |  |  |  |
| *CHI* | *Malus hybrid cultivar* | FJ817485 | Complete |
|  |  |  |  |
| *C4H* | *Malus x domestica* | [DQ075002](http://www.ncbi.nlm.nih.gov/entrez/query.fcgi?cmd=Retrieve&db=Nucleotide&list_uids=68164960&dopt=GenBank&RID=CV0EHFMF01S&log$=nuclalign&blast_rank=1) | Complete |
|  |  |  |  |
| *CHS* | *Malus x domestica* | EU872157 | Complete |
|  |  |  |  |
| *DFR* | [*Crataegus monogyna*](http://www.ncbi.nlm.nih.gov/Taxonomy/Browser/wwwtax.cgi?id=140997) | AY786995 | Complete |
|  |  |  |  |
| *LAR* | [*Malus x domestica*](http://www.ncbi.nlm.nih.gov/Taxonomy/Browser/wwwtax.cgi?id=3750) | DQ139836 | Complete |
|  |  |  |  |
| *F3H* | *Malus hybrid cultivar* | FJ817486 | Complete |
|  |  |  |  |
| *PAL* | *Pyrus communis* | DQ230992 | Complete |
