# Supplementary material for: Designing universal primers for the isolation of DNA sequences encoding Proanthocyanidins biosynthetic enzymes in Crataegus aronia
Source: BMC Res Notes. 2012 Aug 10;5:427. doi: 10.1186/1756-0500-5-427 (PMC3492024; doi:10.1186/1756-0500-5-427)
Supplement: Additional file 2 — Multiple sequence alignment analysis of the ANS gene showing the position of the ANS designed primers (underlined) used in this study. [file 1756-0500-5-427-S2.docx]

Additional File 2. Multiple sequence alignment analysis of the *ANS* gene showing the position of the *ANS* designed primers (underlined) used in this study.

300 310 320 330 340 350

| | | | | |

*P. communis* DQ230994 GAAGGCCGGTAAGGCCTTCTTTGACCTTCCCATTGAGCAGAAGGAGAAGTA

*M. domestica ANS* contig GAAGGCCGGTAAGGCCTTCTTTGACCTTCCCATTGAGCAGAAGGAGAAGTA

*P. armeniaca ANS* contig GAAGGCCGGGAAGGCCTTTTTCGATCTTCCCATTGAGCAAAAGGAGAAGTA

*F. vesca ANS* contig GAAGGCCGGAAAAGCCTTCTTTGATCTTCCCATTGAGCAGAAGGAGAAGTA

*F. ananassa* AY695817 GAAGGCCGGAAAAGCCTTCTTTGATCTTCCCATTGAGCAGAAGGAGAAGTA

*P. cerasifera* EF683132 GAAGGCCGGGAAGGCCTTTTTCGATCTTCCCATTGAGCAAAAGGAGAAGTA

*P. persica ANS* contig GAAGGCCGGGAAGGCCTTTTTCGATCTTCCCATTGAGCAAAAGGAGAAGTA

*R. hybrid ANS* contig GAAGGCCGGACAAGCCTTCTTTGATCTTCCCATTGAGCAGAAGGAGAAGTA

**ANSFwd1** TTTGAYCTTCCCATTGAGCA

350 360 370 380 390 400

| | | | | |

*P. communis* DQ230994 ACCAGGCCTCTGGTAAGATTCAAGGCTATGGAAGCAAGCTTGCAAACAATG

*M. domestica ANS* contig ACCAGGCCTCTGGTAAGATTCAAGGCTATGGAAGCAAGCTTGCAAACAATG

*P. armeniaca ANS* contig ACCAGGCCTCTGGCAAAATTCAAGGCTATGGAAGCAAGCTAGCAAACAATG

*F. vesca ANS* contig ATTAGGCCTCCGGCAAAATTCAAGGCTACGGAAGCAAGCTAGCAAACAATG

*F. ananassa* AY695817 ACCAGGCCTCCGGCAAAATTCAAGGCTACGGAAGCAAGCTAGCAAACAATG

*P. cerasifera* EF683132 ACCAGGCCTCTGGCAAAATTCAAGGCTATGGAAGCAAGCTAGCAAACAATG

*P. persica ANS* contig ACCAGGCCTCTGGCAAAATTCAAGGCTATGGAAGCAAGCTAGCAAACAATG

*R. hybrid ANS* contig ACCAGGCCTCGGGCAAAATTCAAGGCTACGGAAGCAAGCTAGCAAACAATG

400 410 420 430 440 450

| | | | | |

*P. communis* DQ230994 GCATCTGGGCAGCTTGAGTGGGAGGACTACTTCTTCCACTGTGTATACCCA

*M. domestica ANS* contig GCATCTGGGCAGCTTGAGTGGGAGGACTACTTCTTCCACTGTGTATACCCA

*P. armeniaca ANS* contig GCTTCTGGGCAGCTTGAGTGGGAGGACTACTTCTTCCACCTTGTTTACCCT

*F. vesca ANS* contig GCTTCCGGCCAACTTGAGTGGGAGGACTACTTTTTCCACTGTGTTTATCCT

*F. ananassa* AY695817 GCTTCCGGCCAACTTGAGTGGGAGGACTACTTTTTCCACTGTGTATATCCT

*P. cerasifera* EF683132 GCCTCTGGGCAGCTTGAGTGGGAGGACTACTTCTTCCACCTTGTATACCCT

*P. persica ANS* contig GCTTCTGGGCAGCTTGAGTGGGAGGACTACTTCTTCCACCTTGTATACCCT

*R. hybrid ANS* contig GCTTCCGGGCAACTTGAGTGGGAGGACTATTTTTTCCACTGTGTATATCCT

**ANSFwd2** CAGCTTGAGTGGGAGGACTA

630 640 650 660 670 680

| | | | | |

*P. communis* DQ230994 AGAGCTCCTCTTGCAAATGAAAATCAACTACTACCCAAAATGCCTCAGCCG

*M*. *domestica* contig AGAGCTCCTCTTGCAAATGAAAATCAACTACTACCCAAAATGCCTCAGCCG

*P*. armeniaca contig AGAGCTTCTCTTGCAAATGAAAATCAACTACTACCCAGTTTGCCTCAGCCA

*F*. *vesca* contig AGAACTCCTCCTGCAAATGAAGATCAACTACTACCCAAAATGCCTCAGCCG

*F*. *ananassa* AY695817 AGAACTCCTCCTGCAAATGAAGATCAACTACTACCCAAAATGCCTCAGCCG

*P*. *cerasifera* EF683132 GGAGCTTCTCTTGCAAATGAAAATCAACTACTACCCAGTTTGCCTCAGCCA

*P*. *persica* ANS contig GGAGCTTCTCTTGCAAATGAAAATCAACTACTACCCACTTTGCCTCAGCCA

*R*. *hybrid* ANS contig AGAACTCCTCCTGCAAATGAAAATCAACTACTACCCAAAATGCCTCAGCCG

**ANSRev1** TCAACTACTACCCAAAATGC

680 690 700 710 720 730

| | | | | |

*P. communis* DQ230994 GCTTGCACTTGGTGTTGAAGCTCACACTGACGTGAGTGCACTCACTTCATC

*M*. *domestica* contig GCTTGCACTTGGTGTTGAAGCTCACACTGACGTGAGTGCACTCACTTCATC

*P*. *armeniaca* contig GCTTGCACTTGGTGTTGAAGCTCACACTGATGTCAGTGCACTCACTTCATA

*F*. *vesca* contig ACTTGCACTCGGCGTGGAAGCTCATACAGACATAAGTGCACTCACTTCATC

*F*. *ananassa* AY695817 ACTTGCACTCGGCGTGGAAGCTCATACAGACATAAGTGCACTCACTTCATC

*P*. *cerasifera* EF683132 GCTTGCACTTGGTGTTGAAGCTCACACTGATGTCAGTGCACTCACTTCATA

*P*. *persica* ANS contig GCTTGCACTTGGTGTCGAAGCTCACACTGATGTCAGTGCACTCACTTCATA

*R*. *hybrid* ANS contig ACTTGCCCTCGGCGTGGAAGCCCACACTGACATAAGTGCACTCACTTCATC

730 740 750 760 770 780

| | | | | |

*P. communis* DQ230994 CCTCCACAACATGGTTCCTGGCCTGCAGCTTTTCTATGAAGGAAAGTGGGT

*M*. *domestica* contig CCTCCACAACATGGTTCCTGGCCTGCAGCTTTTCTATGAAGGAAAGTGGGT

*P*. *armeniaca* contig ACTCCACAACATGGTTCCTGGCCTGCAGCTTTTCTATGAAGGCAAATGGGT

*F*. *vesca* contig CCTCCACAACATGGTTCCCGGCCTGCAGCTCTTCTACGGCGGCAAATGGGT

*F*. *ananassa* AY695817 CCTCCACAACATGGTTCCCGGCCTGCAGCTCTTCTACGGCGGCAAATGGGT

*P*. *cerasifera* EF683132 ACTCCACAACATGGTTCCTGGCCTGCAGCTTTTCTATGAAGGCAAGTGGGT

*P*. *persica* *ANS* contig ACTCCACAACATGGTTCCTGGCCTGCAGCTTTTCTATGAAGGCAAGTGGGT

*R*. *hybrid* *ANS* contig CCTCCACAACATGGTTCCCGGCCTGCAGCTCTTCTACGGCGGCAAATGGGT

**ANSRev2** CAACATGGTTCCYGGCCTGC
