# Supplementary material for: Designing universal primers for the isolation of DNA sequences encoding Proanthocyanidins biosynthetic enzymes in Crataegus aronia
Source: BMC Res Notes. 2012 Aug 10;5:427. doi: 10.1186/1756-0500-5-427 (PMC3492024; doi:10.1186/1756-0500-5-427)
Supplement: Additional file 3 — Pairwise sequence alignment between the designed primers and the blast retrieved DNA sequences encoding PAs biosynthesis from Malus, Prunus and Fragaria spp. using the NCBI GenBank nucleotide (nr/nt) and est databases. [file 1756-0500-5-427-S3.doc]

Additional file 3. Pairwise sequence alignment between the designed primers and the blast retrieved DNA sequences encoding PAs biosynthesis from *Malus*, *Prunus* and *Fragaria* spp. using the NCBI GenBank nucleotide (nr/nt) and est databases.

| Primer | Organism | Accession number | Pairwise Alignment | e-value |
| --- | --- | --- | --- | --- |
| ANRFwd1 | *Malus spp.* | JN035301.1 | Query 1 CCCACCTMACAGCACTACAA 20  ||||||| ||||||||||||  Sbjct 215 CCCACCTCACAGCACTACAA 234 | 0.001 |
|  | *Prunus spp.* | FC865056.1 | Query 1 CTCACCTMACAGCACTACAA 20  \| \|\|\|\|\| \|\|\|\|\|\|\|\|\|\|\|\|  Sbjct 241 CCCACCTCACAGCACTACAA 260 | 0.13 |
|  | *Fragaria spp.* | DQ251189.1 | Query 1 CCCACCTMACAGCACTACAA 20  \|\|\|\|\|\|\| \|\|\|\|\|\|\|\|\|\|\|\|  Sbjct 152 CCCACCTCACAGCACTACAA 171 | 0.001 |
|  |  |  |  |  |
| ANRRev1 | *Malus spp.* | JN035301.1 | Query 1 TACCGACCAGAAGCAGATTC 20  ||||||||||||||||||||  Sbjct 857 TACCGACCAGAAGCAGATTC 838 | 1e-04 |
|  | *Prunus spp.* | AM288300.1 | Query 1 TACCGACCAGAAGCAGATTC 20  \|\|\|\|\|\|\|\|\|\|\|\|\|\|\|\|\|\|\|\|  Sbjct 168 TACCGACCAGAAGCAGATTC 149 | 0.001 |
|  | *Fragaria spp.* | DQ438979.1 | Query 1 TACCGACCAGAAGCAGATTC 20  \|\|\|\|\|\|\|\|\|\|\|\|\|\|\|\|\|\|\|\|  Sbjct 791 TACCGACCAGAAGCAGATTC 772 | 1e-04 |
|  |  |  |  |  |
| ANRFwd2 | *Malus spp.* | JN035301.1 | Query 1 GGAGTGATTTGGAGTTCTTG 20  ||||||||||||||||||||  Sbjct 521 GGAGTGATTTGGAGTTCTTG 540 | 1e-04 |
|  | *Prunus spp.* | DW346873.1 | Query 1 GGAGTGATTTGGAGTTCTTG 20  \|\|\|\| \|\|\|\|\|\|\|\|\|\|\|\|\|\|\|  Sbjct 200 GGAGGGATTTGGAGTTCTTG 219 | 0.27 |
|  | *Fragaria spp.* | No hit |  |  |
|  |  |  |  |  |
| ANRRev2 | *Malus spp.* | JN035301.1 | Query 1 GGAAAATCTCCAAACTCAGT 20  ||||||||||||||||||||  Sbjct 956 GGAAAATCTCCAAACTCAGT 937 | 1e-04 |
|  | *Prunus spp.* | AM288300.1 | Query 1 GGAAAATCTCCAAACTCAGT 20  \|\|\|\|\|\|\|\|\|\|\|\|\|\|\|\|\|\|\|\|  Sbjct 267 GGAAAATCTCCAAACTCAGT 248 | 0.001 |
|  | *Fragaria spp.* | DQ438979.1 | Query 1 GGAAAATCTCCAAACTCAGT 20  \|\|\|\|\|\|\|\|\|\|\|\|\|\|\|\|\|\|\|\|  Sbjct 890 GGAAAATCTCCAAACTCAGT 871 | 1e-04 |

| Primer | Organism | Accession number | Pairwise Alignment | e-value |
| --- | --- | --- | --- | --- |
| ANSFwd1 | *Malus spp.* | AF117269 | Query 1 TTTGAYCTTCCCATTGAGCA 20  ||||| ||||||||||||||  Sbjct 408 TTTGACCTTCCCATTGAGCA 427 | 0.001 |
|  | *Prunus spp.* | HM543572.1 | Query 1 TTTGAYCTTCCCATTGAGCA 20  \|\| \|\| \|\|\|\|\|\|\|\|\|\|\|\|\|\|  Sbjct 438 TTCGATCTTCCCATTGAGCA 457 | 0.065 |
|  | *Fragaria spp.* | JQ923457.1 | Query 1 TTTGAYCTTCCCATTGAGCA 20  \|\|\|\|\| \|\|\|\|\|\|\|\|\|\|\|\|\|\|  Sbjct 319 TTTGATCTTCCCATTGAGCA 338 | 0.001 |
|  |  |  |  |  |
| ANSRev1 | *Malus spp.* | AF117269 | Query 1 GCATTTTGGGTAGTAGTTGA 20  ||||||||||||||||||||  Sbjct 761 GCATTTTGGGTAGTAGTTGA 742 | 1e-04 |
|  | *Prunus spp.* | ppa007856m* | Query: 1 GCATTTTGGGTAGTAGTTGA 20  \|\|\|\|\|\|\|\|\|\|\|\|\| \|\|\|\|\|\|  Sbjct: 672 GCATTTTGGGTAGAAGTTGA 653 | 0.041 |
|  | *Fragaria spp.* | JQ923457.1 | Query 1 GCATTTTGGGTAGTAGTTGA 20  \|\|\|\|\|\|\|\|\|\|\|\|\|\|\|\|\|\|\|\|  Sbjct 672 GCATTTTGGGTAGTAGTTGA 653 | 1e-04 |
|  |  |  |  |  |
| ANSFwd2 | *Malus spp.* | AF117269 | Query 1 CAGCTTGAGTGGGAGGAYTA 20  ||||||||||||||||| ||  Sbjct 507 CAGCTTGAGTGGGAGGACTA 526 | 0.001 |
|  | *Prunus spp.* | HM543572.1 | Query 1 CAGCTTGAGTGGGAGGAYTA 20  \|\|\|\|\|\|\|\|\|\|\|\|\|\|\|\|\| \|\|  Sbjct 537 CAGCTTGAGTGGGAGGACTA 556 | 0.001 |
|  | *Fragaria spp.* | JQ923457.1 | Query 1 CAGCTTGAGTGGGAGGAYTA 20  \|\| \|\|\|\|\|\|\|\|\|\|\|\|\|\| \|\|  Sbjct 418 CAACTTGAGTGGGAGGACTA 437 | 0.065 |
|  |  |  |  |  |
| ANSRev2 | *Malus spp.* | AF117269 | Query 1 GCAGGCCRGGAACCATGTTG 20  ||||||| ||||||||||||  Sbjct 849 GCAGGCCAGGAACCATGTTG 830 | 0.001 |
|  | *Prunus spp.* | HM543572.1 | Query 1 GCAGGCCRGGAACCATGTTG 20  \|\|\|\|\|\|\| \|\|\|\|\|\|\|\|\|\|\|\|  Sbjct 879 GCAGGCCAGGAACCATGTTG 860 | 0.001 |
|  | *Fragaria spp.* | JQ923457.1 | Query 1 GCAGGCCRGGAACCATGTTG 20  \|\|\|\|\|\|\| \|\|\|\|\|\|\|\|\|\|\|\|  Sbjct 760 GCAGGCCGGGAACCATGTTG 741 | 0.001 |

* the DNA sequence was retrieved from the GDR (www.roseacea.org)

| Primer | Organism | Accession number | Pairwise Alignment | e-value |
| --- | --- | --- | --- | --- |
| 4-CLFwd1 | *Malus spp.* | GO498705.1 | Query 2 CTGGAGAGATTTGCATCAG 20  |||||||||||||||||||  Sbjct 286 CTGGAGAGATTTGCATCAG 304 | 0.004 |
|  | *Prunus spp.* | HE643853.1 | Query 1 CCTGGAGAGATTTGCATCAG 20  \|\|\|\|\|\|\|\|\|\|\|\|\|\|\|\|\| \|\|  Sbjct 534 CCTGGAGAGATTTGCATTAG 553 | 0.068 |
|  | *Fragaria spp.* | HM026180 | Query 1 CCTGGAGAGATTTGCATCAG 20  \|\|\|\|\| \|\|\|\|\|\|\|\|\|\|\|\|\|\|  Sbjct 594 CCTGGTGAGATTTGCATCAG 613 | 0.028 |
|  |  |  |  |  |
| 4-CLRev1 | *Malus spp.* | GO565912.1 | Query 1 AATACTCGATTTATTCTTTTATA 23  |||||||||||||||||||||||  Sbjct 409 AATACTCGATTTATTCTTTTATA 431 | 2e-05 |
|  | *Prunus spp.* | No hit |  |  |
|  | *Fragaria spp.* | AB646330.1 | Query 1 AATACTCGATTTATTCTTTT 20  \|\|\|\|\|\|\| \|\|\|\|\|\|\|\|\|\|\|\|  Sbjct 404 AATACTCTATTTATTCTTTT 385 | 0.38 |
|  |  |  |  |  |
| 4-CLFwd2 | *Malus spp.* | GO530303.1 | Query 1 ATCATGAAAGGTTATCTTAATG 22  ||||||||||||||||||||||  Sbjct 393 ATCATGAAAGGTTATCTTAATG 414 | 1e-04 |
|  | *Prunus spp.* | HE643853.1 | Query 1 ATCATGAAAGGTTATCTTAATG 22*  \|\|\|\|\|\|\|\|\|\|\|\|\|\|\|\|\|\|\|\|\|\|  Sbjct 564 ATCATGAAAGGTTATCTTAATG 585 | 1e-04 |
|  | *Fragaria spp.* | AB646330.1 | Query 1 ATCATGAAAGGTTATCTTAATG 22  \|\|\|\|\|\|\|\|\|\|\|\|\|\| \|\|\|\|\|\|\|  Sbjct 46 ATCATGAAAGGTTACCTTAATG 67 | 0.024 |
|  |  |  |  |  |
| 4-CLRev2 | *Malus spp.* | GO565912.1 | Query 1 GGAATGGCTTCGATGAAAAA 20  ||||||||||||||||||||  Sbjct 391 GGAATGGCTTCGATGAAAAA 410 | 0.001 |
|  | *Prunus spp.* | ppa003747m * | Query: 1 GGAATGGCTTCGATGAAAAA 20  \|\|\|\|\|\|\|\|\|\|\|\|\|\|\|\|\|\|\|\|  Sbjct: 1586 GGAATGGCTTCGATGAAAAA 1567 | 2e-04 |
|  | *Fragaria spp.* | AB646330.1 | Query 1 GGAATGGCTTCGATGAAAAA 20  \|\|\|\|\|\|\|\|\|\|\|\|\|\|\|\|\|\|\|\|  Sbjct 422 GGAATGGCTTCGATGAAAAA 403 | 0.001 |

* the DNA sequence was retrieved from the GDR (www.roseacea.org)

| Primer | Organism | Accession number | Pairwise Alignment | e-value |
| --- | --- | --- | --- | --- |
| CHIFwd1 | *Malus spp.* | FJ817485.1 | Query 1 AGGGGGTYGGAGATTCAGGG 20  ||||||| ||||||||||||  Sbjct 112 AGGGGGTTGGAGATTCAGGG 131 | 0.001 |
|  | *Prunus spp.* | GU990525.1 | Query 1 AGGGGGTYGGAGATTCAGGG 20  \|\|\|\|\|\| \|\|\|\|\|\|\|\|\|\|\|\|  Sbjct 227 AGGGGGCTGGAGATTCAGGG 246 | 0.27 |
|  | *Fragaria spp.* | No hit |  |  |
|  |  |  |  |  |
| CHIRev1 | *Malus spp.* | FJ817485.1 | Query 1 GGGAAGKTTTGATCYTTGAA 20  |||||| ||||||| |||||  Sbjct 440 GGGAAGTTTTGATCTTTGAA 421 | 0.007 |
|  | *Prunus spp.* | GU990525.1 | Query 1 GGGAAGKTTTGATCYTTGAA 20  \|\|\|\|\|\| \|\|\|\|\|\|\| \|\|\|\|\|  Sbjct 555 GGGAAGTTTTGATCCTTGAA 536 | 0.007 |
|  | *Fragaria spp.* | GT150046.1 | Query 1 GGGAAGKTTTGATCYTTGAA 20  \|\|\|\|\|\| \|\|\|\|\|\|\| \|\|\|\|\|  Sbjct 449 GGGAAGGTTTGATCTTTGAA 430 | 0.27 |
|  |  |  |  |  |
| CHIFwd2 | *Malus spp.* | GO514259.1 | Query 1 TTCGTGAAGTTCACGGCGAT 20  ||||||||||||||||||||  Sbjct 178 TTCGTGAAGTTCACGGCGAT 197 | 0.001 |
|  | *Prunus spp.* | GU990525.1 | Query 1 TTCGTGAAGTTCACGGCGAT 20  \|\|\|\|\|\|\|\|\|\|\|\|\|\|\|\|\|\|\|\|  Sbjct 251 TTCGTGAAGTTCACGGCGAT 270 | 1e-04 |
|  | *Fragaria spp.* | AB201755.1 | Query 1 TTCGTGAAGTTCACGGCGAT 20  \|\|\|\|\|\|\|\|\|\|\|\|\|\|\|\|\|\|\|\|  Sbjct 130 TTCGTGAAGTTCACGGCGAT 149 | 1e-04 |
|  |  |  |  |  |
| CHIRev2 | *Malus spp.* | FJ817485.1 | Query 1 TGGGAAGGTCTGATCTTTGA 20  ||||||| | ||||||||||  Sbjct 441 TGGGAAGTTTTGATCTTTGA 422 | 6.9 |
|  | *Prunus spp.* | AM289232.1 | Query 1 TGGGAAGGTCTGATCTTTGA 20  \| \|\|\|\|\|\|\|\|\|\|\|\|\|\|\|\|\|  Sbjct 441 TCGGAAGGTCTGATTTTTGG 422 | 17 |
|  | *Fragaria spp.* | AB201755.1 | Query 1 TGGGAAGGTCTGATCTTTGA 20  \|\|\|\|\|\|\|\|\|\|\|\|\|\|\|\|\|\|\|\|  Sbjct 435 TGGGAAGGTCTGATCTTTGA 416 | 1e-04 |

| Primer | Organism | Accession number | Pairwise Alignment | e-value |
| --- | --- | --- | --- | --- |
| CHIFwd1 | *Malus spp.* | JQ248935.1 | Query 1 CTCCGYATGGGRCAGCGCAA 20  ||||| ||||| ||||||||  Sbjct 285 CTCCGCATGGGGCAGCGCAA 304 | 0.007 |
|  | *Prunus spp.* | HM204478.1 | Query 1 CTCCGYATGGGRCAGCGCAA 20  \|\|\|\|\| \|\|\|\|\| \|\|\|\|\|\|\|\|  Sbjct 211 CTCCGCATGGGGCAGCGCAA 230 | 0.007 |
|  | *Fragaria spp.* | EX676900.1 | Query 1 CTCCGYATGGGRCAGCGCAA 20  \|\|\|\|\| \|\|\|\|\| \|\|\|\|\|\|\|\|  Sbjct 257 CTCCGCATGGGGCAGCGCAA 276 | 0.007 |
|  |  |  |  |  |
| CHIRev1 | *Malus spp.* | JQ248935.1 | Query 1 TTGTTGTACATCATMAGCTG 20  |||||||||||||| |||||  Sbjct 628 TTGTTGTACATCATCAGCTG 609 | 0.001 |
|  | *Prunus spp.* | HM204478.1 | Query 1 TTGTTGTACATCATMAGCTG 20  \|\|\|\|\|\|\|\|\|\|\|\|\|\| \|\|\|\|\|  Sbjct 554 TTGTTGTACATCATCAGCTG 535 | 0.001 |
|  | *Fragaria spp.* | HM026179.1 | Query 1 TTGTTGTACATCATMAGCTG 20  \|\|\|\|\|\|\|\|\|\|\|\|\|\| \|\|\|\|\|  Sbjct 197 TTGTTGTACATCATCAGCTG 178 | 0.001 |
|  |  |  |  |  |
| CHIFwd2 | *Malus spp.* | JQ248935.1 | Query 1 CAGCTBATGATGTACAACAA 20  ||||| ||||||||||||||  Sbjct 609 CAGCTGATGATGTACAACAA 628 | 0.004 |
|  | *Prunus spp.* | HM204478.1 | Query 1 CAGCTBATGATGTACAACAA 20  \|\|\|\|\| \|\|\|\|\|\|\|\|\|\|\|\|\|\|  Sbjct 535 CAGCTGATGATGTACAACAA 554 | 0.004 |
|  | *Fragaria spp.* | HM026179.1 | Query 1 CAGCTBATGATGTACAACAA 20  \|\|\|\|\| \|\|\|\|\|\|\|\|\|\|\|\|\|\|  Sbjct 178 CAGCTGATGATGTACAACAA 197 | 0.004 |
|  |  |  |  |  |
| CHIRev2 | *Malus spp.* | JQ248935.1 | Query 1 TGGATTTCRGGGTGGTTCAC 20  |||||||| |||||||||||  Sbjct 1060 TGGATTTCGGGGTGGTTCAC 1041 | 0.001 |
|  | *Prunus spp.* | HM204478.1 | Query 1 TGGATTTCRGGGTGGTTCAC 20  \|\|\|\|\| \|\| \|\|\|\|\|\|\|\|\|\|\|  Sbjct 986 TGGATCTCAGGGTGGTTCAC 967 | 0.27 |
|  | *Fragaria spp.* | HM02679.1 | Query 1 TGGATTTCRGGGTGGTTCAC 20  \|\|\|\|\|\|\|\| \|\|\|\|\|\|\|\|\|\|\|  Sbjct 630 TGGATTTCGGGGTGGTTCAC 611 | 0.001 |

| Primer | Organism | Accession number | Pairwise Alignment | e-value |
| --- | --- | --- | --- | --- |
| CHSFwd1 | *Malus spp.* | HQ853494.1 | Query 1 AAGGCCATYAAGGAATGGGG 20  |||||||| |||||||||||  Sbjct 443 AAGGCCATCAAGGAATGGGG 462 | 0.001 |
|  | *Prunus spp.* | JN391444.1 | Query 1 AAGGCCATYAAGGAATGGGG 20  \|\|\|\|\|\|\|\| \|\|\|\|\|\|\|\|\|\|\|  Sbjct 1059 AAGGCCATTAAGGAATGGGG 1078 | 0.001 |
|  | *Fragaria spp.* | HM026181.1 | Query 1 AAGGCCATYAAGGAATGGGG 20  \|\|\|\|\|\|\|\| \|\|\|\|\|\|\|\|\|\|\|  Sbjct 320 AAGGCCATTAAGGAATGGGG 339 | 0.001 |
|  |  |  |  |  |
| CHSRev1 | *Malus spp.* | FJ599763.1 | Query 1 AATGTRAGCCCDACTTCACG 20  ||||| ||||| ||||||||  Sbjct 901 AATGTAAGCCCTACTTCACG 882 | 0.029 |
|  | *Prunus spp.* | JN391441.1 | Query 1 AATGTRAGCCCDACTTCACG 20  \|\|\|\|\| \|\|\|\|\| \|\|\|\|\|\|\|\|  Sbjct 1519 AATGTAAGCCCAACTTCACG 1500 | 0.029 |
|  | *Fragaria spp.* | EU024868.1 | Query 1 AATGTRAGCCCDACTTCACG 20  \|\|\|\|\| \|\|\|\|\| \|\|\|\|\|\|\|\|  Sbjct 750 AATGTGAGCCCAACTTCACG 769 | 0.029 |
|  |  |  |  |  |
| CHSFwd2 | *Malus spp.* | HQ853494.1 | Query 1 AAGGAATGGGGHCAGCCCAA 20  ||||||||||| ||||||||  Sbjct 452 AAGGAATGGGGACAGCCCAA 471 | 0.004 |
|  | *Prunus spp.* | JN391444.1 | Query 1 AAGGAATGGGGHCAGCCCAA 20  \|\|\|\|\|\|\|\|\|\|\| \|\|\|\|\|\|\|\|  Sbjct 1068 AAGGAATGGGGCCAGCCCAA 1087 | 0.004 |
|  | *Fragaria spp.* | HM026181.1 | Query 1 AAGGAATGGGGHCAGCCCAA 20  \|\|\|\|\|\|\|\|\|\|\| \|\|\|\|\|\|\|\|  Sbjct 329 AAGGAATGGGGTCAGCCCAA 348 | 0.004 |
|  |  |  |  |  |
| CHSRev2 | *Malus spp.* | HQ853494.1 | Query 1 CTCACYTCRTCCAAAATAAA 20  ||||| || |||||||||||  Sbjct 1158 CTCACCTCGTCCAAAATAAA 1139 | 0.007 |
|  | *Prunus spp.* | JN391444.1 | Query 1 CTCACYTCRTCCAAAATAAA 20  \|\| \|\| \|\| \|\|\|\|\|\|\|\|\|\|\|  Sbjct 1158 CTTACCTCATCCAAAATAAA 1139 | 0.59 |
|  | *Fragaria spp.* | EU024868.1 | Query 1 CTCACYTCRTCCAAAATAAA 20  \|\|\|\|\| \|\| \|\|\|\|\|\|\|\|\|\|\|  Sbjct 5161 CTCACCTCGTCCAAAATAAA 5180 | 0.007 |

| Primer | Organism | Accession number | Pairwise Alignment | e-value |
| --- | --- | --- | --- | --- |
| DFRFwd1 | *Malus spp.* | FJ817487.1 | Query 1 CCTGACGCTGTGGAAGGCGG 20  ||||||||||||||||||||  Sbjct 197 CCTGACGCTGTGGAAGGCGG 216 | 1e-04 |
|  | *Prunus spp.* | HM54357.1 | Query 1 CCTGACGCTGTGGAAGGCGG 20  \|\| \|\|\|\|\|\|\|\|\|\|\|\|\|\|\|  Sbjct 240 ACTTGACGCTGTGGAAGGCT 259 | 0.28 |
|  | *Fragaria spp.* | HM026184.1 | Query 2 CTGACGCTGTGGAAGGCGG 20  \|\|\|\|\|\|\|\|\|\|\|\|\|\|\|\|\|\|\|  Sbjct 124 CTGACGCTGTGGAAGGCGG 142 | 5e-04 |
|  |  |  |  |  |
| DFRRev1 | *Malus spp.* | FJ817487.1 | Query 1 TAATGGTRATGAAATCAATG 20  ||||||| ||||||||||||  Sbjct 591 TAATGGTGATGAAATCAATG 572 | 0.001 |
|  | *Prunus spp.* | HM54357.1 | Query 1 TAATGGTRATGAAATCAATG 20  \|\|\|\|\|\|\| \|\|\|\|\|\|\|\|\|\|\|\|  Sbjct 635 TAATGGTAATGAAATCAATG 616 | 0.001 |
|  | *Fragaria spp.* | AY780884.1 | Query 1 TAATGGTRATGAAATCAATG 20  \|\|\|\| \|\| \|\|\|\|\|\|\|\|\|\|\|\|  Sbjct 571 TAATCGTAATGAAATCAATG 552 | 0.27 |
|  |  |  |  |  |
| DFRFwd2 | *Malus spp.* | FJ817487.1 | Query 1 TGCASCGGAGTGTTYCATGT 20  |||| ||||||||| |||||  Sbjct 258 TGCAGCGGAGTGTTCCATGT 277 | 0.007 |
|  | *Prunus spp.* | HM54357.1 | Query 1 TGCASCGGAGTGTTYCATGT 20  \|\|\|\| \|\|\|\|\|\|\|\|\| \|\|\|\|\|  Sbjct 302 TGCACCGGAGTGTTCCATGT 321 | 0.007 |
|  | *Fragaria spp.* | HM026184.1 | Query 1 TGCASCGGAGTGTTYCATGT 20  \|\|\|\| \|\|\|\|\|\|\|\|\| \|\|\|\|\|  Sbjct 184 TGCACCGGAGTGTTTCATGT 203 | 0.007 |
|  |  |  |  |  |
| DFRRev2 | *Malus spp.* | FJ817487.1 | Query 1 TGAGGCTTGGTGGCATRGATGG 22  |||||||||||||||| |||||  Sbjct 645 TGAGGCTTGGTGGCATGGATGG 624 | 8e-05 |
|  | *Prunus spp.* | HM54357.1.1 | Query 1 TGAGGCTTGGTGGCATRGATGG 22  \|\|\|\|\|\|\|\|\|\|\|\|\|\|\|\| \|\|\|\|\|  Sbjct 689 TGAGGCTTGGTGGCATGGATGG 668 | 8e-05 |
|  | *Fragaria spp.* | AY780884.1 | Query 1 TGAGGCTTGGTGGCATRGATGG 22  \|\|\|\|\|\|\|\|\|\|\|\|\|\|\|\| \|\|\|\|\|  Sbjct 625 TGAGGCTTGGTGGCATAGATGG 604 | 8e-05 |

| Primer | Organism | Accession number | Pairwise Alignment | e-value |
| --- | --- | --- | --- | --- |
| LARFwd1 | *Malus spp.* | DQ139836.1 | Query 1 GTTCGTMGCYGAAGCCAGCC 20  |||||| || ||||||||||  Sbjct 78 GTTCGTAGCTGAAGCCAGCC 97 | 0.007 |
|  | *Prunus spp.* | No hit |  |  |
|  | *Fragaria spp.* | DQ834906.2 | Query 1 GTTCGTMGCYGAAGCCAGCC 20  \|\|\|\|\|\| \|\| \|\|\|\|\|\|\|\|\|\|  Sbjct 81 GTTCGTCGCCGAAGCCAGCC 100 | 0.007 |
|  |  |  |  |  |
| LARRev1 | *Malus spp.* | DQ139837.1 | Query 1 TGGAACYGATCCAACGGTGG 20  |||||| |||||||||||||  Sbjct 557 TGGAACCGATCCAACGGTGG 538 | 0.001 |
|  | *Prunus spp.* | GU938686.1 | Query 1 TGGAACYGATCCAACGGTGG 20  \|\|\|\|\|\| \|\|\|\|\|\|\|\|\|\|\|\|\|  Sbjct 557 TGGAACTGATCCAACGGTGG 538 | 0.001 |
|  | *Fragaria spp.* | DQ087253.1 | Query 1 TGGAACYGATCCAACGGTGG 20  \|\|\|\|\|\| \|\|\|\|\|\|\|\|\|\|\|\|\|  Sbjct 398 TGGAACTGATCCAACGGTGG 379 | 0.001 |
|  |  |  |  |  |
| LARFwd2 | *Malus spp.* | DQ139836.1 | Query 1 AGGGCYRATCCGGTKGAACC 20  ||||| ||||||| |||||  Sbjct 382 AGGGCTGATCCGGTTGAACC 401 | 0.064 |
|  | *Prunus spp.* | GU938686.1 | Query 1 AGGGCYRATCCGGTKGAACC 20  \|\|\|\|\| \|\|\|\|\|\|\| \|\|\|\|\|  Sbjct 382 AGGGCTGATCCGGTGGAACC 401 | 0.064 |
|  | *Fragaria spp.* | DQ087253.1 | Query 2 GGGCYRATCCGGTKGAACC 20  \|\|\|\| \|\|\|\|\|\|\| \|\|\|\|\|  Sbjct 386 GGGCCGATCCGGTGGAACC 404 | 0.300 |
|  |  |  |  |  |
| LARRev2 | *Malus spp.* | DQ139836.1 | Query 1 AGGGTSCGRCCAATTTTCTT 20  ||||| || |||||||||||  Sbjct 740 AGGGTCCGGCCAATTTTCTT 721 | 0.007 |
|  | *Prunus spp.* | GU938686.1 | Query 1 AGGGTSCGRCCAATTTTCTT 20  \|\|\|\|\| \|\| \|\|\|\|\|\|\|\|\|\|  Sbjct 740 AGGGTGCGGACAATTTTCTT 721 | 2.6 |
|  | *Fragaria spp.* | DQ087253.1 | Query 1 AGGGTSCGRCCAATTTTCTT 20  \|\|\|\|\| \|\| \|\|\|\|\|\|\|\|\|\|\|  Sbjct 581 AGGGTGCGACCAATTTTCTT 562 | 0.007 |

| Primer | Organism | Accession number | Pairwise Alignment | e-value |
| --- | --- | --- | --- | --- |
| F3HFwd1 | *Malus spp.* | FJ817486.1 | Query 1 GACATGTCCGGYGGCAAAAAGGG 23  ||||||||||| |||||||||||  Sbjct 371 GACATGTCCGGTGGCAAAAAGGG 393 | 2e-05 |
|  | *Prunus spp.* | HM543570.1 | Query 1 GACATGTCCGGYGGCAAAAAGGG 23  \|\|\|\|\|\|\|\|\|\|\| \|\|\|\|\|\|\|\|\|\|\|  Sbjct 383 GACATGTCCGGCGGCAAAAAGGG 405 | 2e-05 |
|  | *Fragaria spp.* | AY691919.1 | Query 1 GACATGTCCGGYGGCAAAAAGGG 23  \|\|\|\|\|\|\|\|\|\|\| \|\|\|\|\|\|\|\|\|\|\|  Sbjct 761 GACATGTCCGGTGGCAAAAAGGG 783 | 2e-05 |
|  |  |  |  |  |
| F3HRev1 | *Malus spp.* | FJ817486.1 | Query 1 CTCATCTTCTTCTTGTACAT 20  ||||||||||||||||||||  Sbjct 1044 CTCATCTTCTTCTTGTACAT 1025 | 1e-04 |
|  | *Prunus spp.* | HM543570.1 | Query 2 TCATCTTCTTCTTGTACAT 20  \|\|\|\|\|\|\|\|\|\|\|\|\|\|\|\|\|\|\|  Sbjct 1055 TCATCTTCTTCTTGTACAT 1037 | 5e-04 |
|  | *Fragaria spp.* | AY691919.1 | Query 1 CTCATCTTCTTCTTGTACAT 20  \|\|\|\|\|\|\|\|\|\|\|\|\|\|\|\|\|\|\|\|  Sbjct 2103 CTCATCTTCTTCTTGTACAT 2084 | 1e-04 |
|  |  |  |  |  |
| F3HFwd2 | *Malus spp.* | FJ817486.1 | Query 1 ACGTGGATCACCGTTCAACC 20  ||||||||||||||||||||  Sbjct 788 ACGTGGATCACCGTTCAACC 807 | 1e-04 |
|  | *Prunus spp.* | HM543570.1 | Query 1 ACGTGGATCACCGTTCAACC 20  \|\|\|\|\|\|\|\|\|\|\|\|\|\|\|\|\|\|\|\|  Sbjct 800 ACGTGGATCACCGTTCAACC 819 | 1e-04 |
|  | *Fragaria spp.* | AY691919.1 | Query 1 ACGTGGATCACCGTTCAACC 20  \|\|\|\|\|\|\|\|\|\|\|\|\|\|\|\|\|\|\|\|  Sbjct 1335 ACGTGGATCACCGTTCAACC 1354 | 1e-04 |
|  |  |  |  |  |
| F3HRev2 | *Malus spp.* | FJ817486.1 | Query 1 GGTTGAACGGTGATCCACGT 20  ||||||||||||||||||||  Sbjct 807 GGTTGAACGGTGATCCACGT 788 | 1e-04 |
|  | *Prunus spp.* | HM543570.1 | Query 1 GGTTGAACGGTGATCCACGT 20  \|\|\|\|\|\|\|\|\|\|\|\|\|\|\|\|\|\|\|\|  Sbjct 819 GGTTGAACGGTGATCCACGT 800 | 1e-04 |
|  | *Fragaria spp.* | AY691919.1 | Query 1 GGTTGAACGGTGATCCACGT 20  \|\|\|\|\|\|\|\|\|\|\|\|\|\|\|\|\|\|\|\|  Sbjct 1354 GGTTGAACGGTGATCCACGT 1335 | 1e-04 |

| Primer | Organism | Accession number | Pairwise Alignment | e-value |
| --- | --- | --- | --- | --- |
| PALFwd1 | *Malus spp.* | GO503112.1 | Query 1 ATCGATGTTTCRAGGAACAA 20  ||||||||||| ||||||||  Sbjct 44 ATCGATGTTTCAAGGAACAA 63 | 0.007 |
|  | *Prunus spp.* | GO499681.1 | Query 1 ATCGATGTTTCRAGGAACAA 20  \|\|\|\|\|\|\|\|\|\|\| \|\|\|\|\|\|\|\|  Sbjct 353 ATCGATGTTTCGAGGAACAA 372 | 0.007 |
|  | *Fragaria spp.* | HM641823.1 | Query 1 ATCGATGTTTCRAGGAACAA 20  \|\|\|\|\|\|\|\| \|\| \|\|\|\|\|\|\|\|  Sbjct 1342 ATCGATGTCTCAAGGAACAA 1361 | 0.27 |
|  |  |  |  |  |
| PALRev1 | *Malus spp.* | GO535001.1 | Query 1 AAAGAGTTAACATCTTGGTT 20  ||||||||||||||||||||  Sbjct 65 AAAGAGTTAACATCTTGGTT 46 | 0.001 |
|  | *Prunus spp.* | GO497578.1 | Query 1 AAAGAGTTAACATCTTGGTT 20  \|\|\|\|\|\|\|\|\|\|\|\|\|\|\|\|\|\|\|\|  Sbjct 168 AAAGAGTTAACATCTTGGTT 149 | 0.001 |
|  | *Fragaria spp.* | No hit |  |  |
|  |  |  |  |  |
| PALRev2 | *Malus spp.* | JQ248934.1 | Query 1 AAAAATGTRGAAGACATGAG 20  |||||||| |||||||||||  Sbjct 1559 AAAAATGTGGAAGACATGAG 1540 | 0.001 |
|  | *Prunus spp.* | JN815262.1 | Query 1 AAAAATGTRGAAGACATGAG 20  \|\|\|\|\|\|\|\| \|\|\|\|\|\|\|\|\|\|\|  Sbjct 351 AAAAATGTGGAAGACATGAG 332 | 0.001 |
|  | *Fragaria spp.* | AB360393.1 | Query 1 AAAAATGTRGAAGACATGAG 20  \|\|\|\|\|\|\|\| \|\|\|\|\|\|\|\|\|\|\|  Sbjct 104 AAAAATGTGGAAGACATGAG 85 | 0.001 |
