# Supplementary material for: Designing universal primers for the isolation of DNA sequences encoding Proanthocyanidins biosynthetic enzymes in Crataegus aronia
Source: BMC Res Notes. 2012 Aug 10;5:427. doi: 10.1186/1756-0500-5-427 (PMC3492024; doi:10.1186/1756-0500-5-427)
Supplement: Additional file 4 — Estimated sizes of PCR amplicons of the PAs biosynthesis gene resulted from using different primers combinations and cDNA prepared from Crataegus callus. [file 1756-0500-5-427-S4.docx]

Additional file 4. Estimated sizes of PCR amplicons of the PAs biosynthesis gene resulted from using different primers combinations and cDNA prepared from *Crataegus* callus.

| Gene name | Primers combination | Expected size (bp) ^a^ | PCR amplicon size (bp) |
| --- | --- | --- | --- |
| *ANR* | ANRFwd1 + ANRRev1^*^ | 643 | 650 |
|  | ANRFwd1 + ANRRev2 | 742 | N.D.^b^ |
|  | ANRFwd2 + ANRRev2 | 436 | 450 |
|  | ANRFwd2 + ANRRev1 | 337 | 350 |
|  |  |  |  |
| ***ANS*** | ANSFwd1 + ANSRev1 | 441 | 500 |
|  | ANSFwd1 + ANSRev2 ^*^ | 453 | 460 |
|  | ANSFwd2 + ANSRev2 | 343 | N.D. |
|  | ANSFwd2 + ANSRev1 | 255 | N.D. |
|  |  |  |  |
| *4-CL* | 4-CLFwd1 + 4-CLRev1 | 389 | 400 |
|  | 4-CLFwd1 + 4-CLRev2 | 407 | 400 |
|  | 4-CLFwd2 + 4-CLRev2 ^*^ | 377 | 380 |
|  | 4-CLFwd2 + 4-CLRev1 | 359 | 360 |
|  |  |  |  |
| *CHI* | CHIFwd1 + CHIRev1 | 329 | 400 |
|  | CHIFwd1 + CHIRev2 | 330 | N.D. |
|  | CHIFwd2 + CHIRev2 | 306 | 330 |
|  | CHIFwd2 + CHIRev1 ^*^ | 305 | 300 |
|  |  |  |  |
| *C4H* | C4HFwd1 + C4HRev1 ^*^ | 344 | 350 |
|  | C4HFwd1 + C4HRev2 | 766 | 800 |
|  | C4HFwd2 + C4HRev2 | 437 | 450 |
|  | C4HFwd2 + C4HRev1 | N.E^c^ | - |
|  |  |  |  |
| *CHS* | CHSFwd1 + CHSRev1 | 461 | 500 |
|  | CHSFwd1 + CHSRev2 | 716 | 750 |
|  | CHSFwd2 + CHSRev2 | 707 | 750 |
|  | CHSFwd2 + CHSRev1 ^*^ | 452 | 460 |
|  |  |  |  |
| *DFR* | DFRFwd1 + DFRRev1 | 395 | 400 |
|  | DFRFwd1 + DFRRev2 | 449 | 450 |
|  | DFRFwd2 + DFRRev2 ^*^ | 388 | 400 |
|  | DFRFwd2 + DFRRev1 | 334 | 450 |
|  |  |  |  |
| *LAR* | LARFwd1 + LARRev1 | 480 | N.D. |
|  | LARFwd1 + LARRev2 | 663 | N.D. |
|  | LARFwd2 + LARRev2 ^*^ | 359 | 360 |
|  | LARFwd2 + LARRev1 | 176 | 200 |
|  |  |  |  |
| *F3H* | F3HFwd1 + F3HRev1 | 674 | 650 |
|  | F3HFwd1 + F3HRev2 ^*^ | 437 | 450 |
|  | F3HFwd2 + F3HRev2 | 257 | N.D. |
|  | F3HFwd2 + F3HRev1 | N.E | - |
|  |  |  |  |
| *PAL* | PALFwd1 + PALRev1 | 320 | 320 |
|  | PALFwd1 + PALRev2 ^*^ | 389 | 400 |
|  |  |  |  |

^a^ Expected size of PCR amplicons anticipated using the corresponding reference gene DNA sequence.

^b^ No amplicon was detected in the PCR.

^c^ No amplicon was expected in the PCR.

^*^ Most successful primers combinations in amplifying PCR products and used in subsequent analysis.
